# An ecological study of chronic kidney disease in five Mesoamerican countries: associations with crop and heat

**DOI:** 10.1186/s12889-021-10822-9

**Published:** 2021-05-01

**Authors:** Erik Hansson, Ali Mansourian, Mahdi Farnaghi, Max Petzold, Kristina Jakobsson

**Affiliations:** 1 School of Public Health and Community Medicine, Institute of Medicine, University of Gothenburg, Gothenburg, Sweden; 2 La Isla Network, Washington, D.C., USA; 3 GIS Centre, Department of Physical Geography and Ecosystem Science, Lund University, Lund, Sweden; 4 Occupational and Environmental Medicine, Sahlgrenska University Hospital, Gothenburg, Sweden

**Keywords:** Chronic kidney disease (CKD), Mesoamerican nephropathy (MeN), Chronic kidney disease of non-traditional origin (CKDnt), Chronic kidney disease of uncertain etiology (CKDu), Heat stress, Agriculture, Occupation

## Abstract

**Background:**

Mesoamerica is severely affected by an epidemic of Chronic Kidney Disease of non-traditional origin (CKDnt), an epidemic with a marked variation within countries. We sought to describe the spatial distribution of CKDnt in Mesoamerica and examine area-level crop and climate risk factors.

**Methods:**

CKD mortality or hospital admissions data was available for five countries: Mexico, Guatemala, El Salvador, Nicaragua and Costa Rica and linked to demographic, crop and climate data. Maps were developed using Bayesian spatial regression models. Regression models were used to analyze the association between area-level CKD burden and heat and cultivation of four crops: sugarcane, banana, rice and coffee.

**Results:**

There are regions within each of the five countries with elevated CKD burden. Municipalities in hot areas and much sugarcane cultivation had higher CKD burden, both compared to equally hot municipalities with lower intensity of sugarcane cultivation and to less hot areas with equally intense sugarcane cultivation, but associations with other crops at different intensity and heat levels were not consistent across countries.

**Conclusion:**

Mapping routinely collected, already available data could be a first step to identify areas with high CKD burden. The finding of higher CKD burden in hot regions with intense sugarcane cultivation which was repeated in all five countries agree with individual-level studies identifying heavy physical labor in heat as a key CKDnt risk factor. In contrast, no associations between CKD burden and other crops were observed.

**Supplementary Information:**

The online version contains supplementary material available at 10.1186/s12889-021-10822-9.

## Background

Over the past two decades, there has been increasing recognition of widespread chronic kidney disease (CKD) in rural working-age populations in some parts of the world, notably Mesoamerica [1]. This cannot be explained by traditional causes such as hypertension or diabetes [2], nor any other known cause, giving the disease the acronym CKDnt - *nt* for non-traditional origin.

Previous studies find high spatial variation in CKD burden within Mesoamerican countries affected by CKDnt (Costa Rica [3], Guatemala [4] and El Salvador [5, 6]) and relate this variation to differences in altitude and agricultural practices, with low-land areas with much sugarcane cultivation being more affected [3–5]. One study also identified cultivation of other crops; cotton, coffee, corn and beans as an area level risk factor, albeit at a smaller extent [6]. The former finding is congruent with the high rate of kidney injury [7–15] and high prevalence of CKDnt among sugarcane workers in coastal regions [5, 16], but not in highland areas [5], which has led to the hypothesis that CKDnt is caused by repeated excessive heat exposure [17, 18]. The latter finding of associations with other crops was interpreted as in line with another hypothesis of the underlying cause of CKDnt - pesticide exposure [6].

Area-level associations between crop types and CKD could arise from effects of internal heat production during strenuous manual labor in industrial agriculture, occupational and non-occupational agrochemical exposure, that certain crops might preferably be grown in certain soils associated with drinking water metal contamination, the combination of these, or several other possible mechanisms. These possible effects are difficult to separate in an ecological study, especially as information on physical efforts of workers (such as degree of mechanization) and agrochemical use is fragmented and largely unavailable. Environmental heat is an important determinant of total heat exposure, as it determines at what level of internal heat production (i.e. physical effort) body temperature becomes elevated, and it also likely affects how agrochemicals are used to control pests, as pests vary by climate zones. An understanding of which crops are associated with CKDnt and at what heat levels could help prioritizing hypotheses on which agricultural practices are associated with CKDnt.

The role of occupational heat stress versus a hitherto unidentified toxin, such as a pesticide, as a main driver of the CKDnt epidemic is debated [19, 20]. Identifying crops that consistently, either at all temperatures or only in high heat are associated with CKD in Mesoamerica could help direct further studies. Prevalence studies can be directed to capture contrasts in likely exposure to these crops and crop-heat combinations, verifying differences in CKD burden, and the associated occupational and environmental exposure between crop and crop-heat combinations can be described in detail and compared.

### Aims

The aim of the present study is to improve understanding of the spatial heterogeneity of CKD burden, and hence indirectly CKDnt, across Mesoamerica. As a first objective, we map modelled estimates of CKD mortality or hospital admissions. As a second objective, we describe how CKD burden, *within each country,* is related to environmental heat and *relative* intensity of agroindustrial crop type (sugarcane, rice, coffee, and banana), and the interaction between heat and crop intensity. Thus, we seek to identify crops which consistently are associated with an increased risk of elevated CKD burden at all cultivation temperatures or only at high ambient temperatures.

It should be stressed already at this stage that we are aiming at understanding *within-country* CKD *burden* and exposure associations as opposed to *absolute* levels of CKD, or *exact* dose-response relationships between explanatory factors and CKD. We use the more generic term *burden* to indicate how affected a geographical area of a country is by CKD compared to other areas in the same country rather than mortality ratio, as this cannot be estimated in the data available for all countries. As available data are not directly comparable between countries we cannot and do not combine countries into the same statistical *analysis*. We nevertheless consider it important to include as many countries as possible when *presenting* the results, as consistencies and differences in the spatial distribution of CKD, and association between crops, heat and CKD between countries can point to important causal mechanisms. This is analogous to a review *presenting* the results of several studies, but refraining from a formal meta-*analysis* if differences in study design do not allow for this.

## Methods

### Outcome data

National statistics departments and health ministry web-pages for each of the Mesoamerican countries were examined for municipality-level information on CKD deaths. Mexico [21] and Guatemala [22] had the full mortality registry available online at their statistic department webpages, and Nicaragua had number of deaths due to the top 15 causes of deaths per municipality per year at their health ministry webpage [23], while data from the remaining countries could not be accessed online. E-mails were sent to health ministries, statistics departments, and researchers if there were previous useful publications, and personal contacts were taken at a research workshop [24]. Through these routes, the rate of hospital admissions due to un-specified chronic kidney disease by municipality could be obtained for El Salvador [6], and the number of CKD deaths by age, sex and municipality from Costa Rica [3] (Table 1). From Panama only data for one year was available, which was considered too little to produce meaningful estimates, and no data could be obtained from Honduras or Belize. CKD hospital admissions rate per municipality in El Salvador was obtained through contact with the authors of a previous publication [6]. This rate was converted to number of cases by multiplying it with the population size from the census data originally used for estimation of the rate [6, 25]. This yielded integer numbers in the great majority of municipalities, but the total sum was almost twice as large as the sum reported in the main text in the original publication [6]. There was however good geographical agreement with the distribution reported in the original publication, which is the most important for the present analysis.

**Table 1:** Outcome data sources

Country	Data type	Data source	Additional outcome event information		Administrative area analysis level	Temporal extent	Number of analysis area units
			Age	Sex			
Mexico	Deaths	Mortality registry [21]	Yes	Yes	Second (Municipality)	2010–2016	2456
Guatemala	Deaths	Mortality registry [22]	Yes	Yes	Second (Municipality)	2009–2015	333
El Salvador	Hospital admissions	Published study [6]	No	No	Second (Municipality)	2005–2010	262
Nicaragua	Deaths	MINSA reports [23]	No	No	Second (Municipality)	2016–2018	153
Costa Rica	Deaths	Published study [3]	Yes	Yes	Second (Canton)	2004–2012	81

*MINSA* Ministerio de Salud = Ministry of Health

### Independent variables

A detailed account of the methods used for management of spatial datasets is available in Supplement A. In each country, the administrative units used cover the entire country.

#### Main exposure variables

##### Crops

Current crop cultivation data was obtained online from agricultural censuses, land use maps or production records (Table 2), assigned to each municipality (Supplement A), and stratified to two crop-intensity strata; high (top 20%) and low (bottom 80%), for each country separately. This division yielded a suitable minimum number of municipalities in each category except for Mexico, which has a large number of municipalities which completely lack cultivation of the crops of interest. Here, the categories 5 and 95% were used instead.

**Table 2:** Explanatory variable data sources

Variable	Spatial domain	Temporal domain	Data source
Population density	All countries	2010	WorldPop [26]
Ambient temperature	All countries	1970–2010	Worldclim [27, 28]
Crop cultivation	Mexico	2009–2012	Mexican government productivity data [29]
	Guatemala	2010	Guatemalan government land use map [30]
		2011–2013	Rice productivity [22]
	El Salvador	2007–2008	Agricultural Census [6]
	Nicaragua	2016	Sugarcane only: Land use map [31]
		2011	Agricultural census [32]
	Costa Rica	2014	Agricultural census [33] and land use maps [34] for sugarcane

##### Heat

A raster map of modelled monthly maximum ambient temperature (T_max_) [27, 28] was used to estimate T_max_ for each municipality. The mean maximum ambient over the whole year was dichotomized to above and below 30 °C.

#### Confounders

Population density was identified as potentially confounding a relation between crop cultivation and death or hospitalization attributed to kidney disease, and we therefor sought to adjust for this.

Population density estimates [26] was assigned to each municipality as described in Supplement A. Population density was categorized to bottom quartile, within interquartile range and top quartile within each country.

### Statistical analysis

A detailed account of the methods are presented in Supplement B, while the general approach is described below.

The differences in which types of data was available between countries (Table 1) necessitated different approaches for each country. If data was available, we modelled the age-standardized CKD mortality rate for working-age (20–50 years in Costa Rica and Guatemala, 18–60 years in Mexico) men and women separately using Poisson regression, but exceptions had to be made for Nicaragua (overall proportional mortality modelled using logistic regression) and El Salvador (overall admission rate modelled using Poisson regression). For Nicaragua, the proportion of non-communicable disease (NCD) deaths that were due to CKD was modelled as a way of adjusting for age (as CKD and NCD deaths normally occur in the same age strata) in the absence of information on an age distribution of deaths.

For the first objective, descriptive mapping of the distribution of within-country CKD burden, Bayesian spatial regression models were used to obtain smoothed municipality-level odds or rate ratios.

For the second objective of identifying crops which consistently were associated with high CKD burden at all heat levels or just in high heat, we used regression modelling to explore associations between an interaction between the crop cultivation density and heat variable, and CKD burden. In this assessment we used both “standard” frequentist non-spatial regression modelling and Bayesian spatial regression modelling as spatial regression models may obscure important relationships between spatially correlated explanatory factors and outcome variables [35, 36]. All models were adjusted for the categorical population density variable. For each country, regression models with each of the four crop density variables separately (banana, sugarcane, coffee and rice) and their interaction with heat were estimated. A municipality can have intense sugarcane cultivation and low intensity coffee cultivation, and may thus be in the intense cultivation category in the sugarcane analysis but the no-intense category in the coffee analysis.

**Non-spatial Poisson and logistic regression models** were estimated in Stata v15 using the *poisson* and *fracreg logit* commands for Poisson and logistic regression respectively.

**Spatial Bayesian Poisson and logistic regression models** were estimated using WinBUGS [37], which offer built-in features for Bayesian regression modelling. We used a Gaussian conditional autoregressive prior structure for the spatially correlated effects, with municipalities sharing a corner or border considered adjacent, and included also spatially uncorrelated random effects with a normally distributed prior structure.

Spatially correlated random effects smooth municipality-level effect estimates towards its neighboring municipalities, and spatially uncorrelated random effects smooth municipality-level effect estimates towards the overall mean, reducing the impact of random fluctuations in low-population areas often dominating raw data maps [36]. Spatially correlated random effects also account for spatial autocorrelation, which if neglected risk leading to biased estimates with overly narrow confidence intervals [36].

After manually assessing convergence models were run to obtain samples from the posterior distribution. The sum of the spatially uncorrelated and correlated random effects for each municipality was translated to the CKD burden rate ratio or odds ratio scale to obtain relative municipality-level CKD burden. The median and 95% range was used as main estimate and 95% Credibility Interval (CrI).

## Results

The proportion of municipalities with intense cultivation of each of the crops above 30 °C varied between countries and crops. Generally, municipalities with intense coffee cultivation were rarely hot (0–12%), whereas the proportion that were hot among the municipalities with intense cultivation of the other crops was higher and more mixed between countries (Tables 3 and 4).

**Table 3:** Descriptive data for Mexico, Guatemala and Costa Rica

		Mexico				Guatemala				Costa Rica			
		CKD deaths^a^ (Obs/Exp)	EE^b^	Municipalities		CKD deaths^a^ (Obs/Exp)	EE^b^	Municipalities		CKD deaths^a^ (Obs/Exp)	EE^b^	Cantons	
				N	%			N	%			N	%
Population density quartile	1	2245/1633	ref	614	25%	434/323	ref	83	25%	86/51	ref	20	25%
	2–3	3494/3525	0.7	1228	50%	534/535	0.7	166	50%	104/105	0.6	40	50%
	4	7279/7861	0.8	614	25%	554/667	0.6	84	25%	73/107	0.4	21	25%
Crop and cultivation intensity	T_max_												
Banana													
Low	< 30°	9975/9871	ref	1784	73%	903/1235	ref	272	82%	103/166	ref	47	58%
	> 30°	2264/2541	0.9	550	22%	277/160	2.4	40	12%	118/30	6.3	18	22%
High	< 30°	235/234	1.0	71	3%	4/7	0.8	2	1%	38/56	1.1	13	16%
	> 30°	545/373	1.4	51	2%	338/120	3.9	19	6%	4/11	0.6	3	4%
Rice													
Low	< 30°	9960/9866	ref	1808	74%	824/1113	ref	218	65%	132/216	ref	57	70%
	> 30°	2115/2461	0.9	529	22%	523/237	3.0	49	15%	29/18	2.6	9	11%
High	< 30°	250/240	1.0	47	2%	83/129	0.9	56	17%	9/6	2.5	3	4%
	> 30°	694/453	1.5	72	3%	92/43	2.9	10	3%	93/23	6.6	12	15%
Sugarcane													
Low	< 30°	9938/9879	ref	1794	73%	803/1149	ref	245	74%	118/193	ref	51	63%
	> 30°	2292/2679	0.9	542	22%	124/128	1.4	22	7%	40/21	3.1	14	17%
High	< 30°	272/226	1.2	61	2%	104/93	1.6	29	9%	23/29	1.3	9	11%
	> 30°	517/235	2.2	59	2%	491/152	4.6	37	11%	82/20	6.7	7	9%
Coffee													
Low	< 30°	9889/9799	ref	1740	71%	764/1065	ref	216	65%	88/142	ref	44	54%
	> 30°	2761/2889	0.9	595	24%	562/245	3.2	51	15%	122/41	4.8	21	26%
High	< 30°	321/306	1.0	115	5%	143/177	1.1	58	17%	53/80	1.1	16	20%
	> 30°	48/25	1.9	6	0%	53/35	2.1	8	2%	−/−	–	0	0%

^a^Number of deaths male 20–50 year olds in Costa Rica and Guatemala, 18–60 years in Mexico. ^b^Age-adjusted mortality ratio. *CKD *Chronic Kidney Disease, *Obs* observed, *Exp* expected, *EE* effect estimate

**Table 4:** Descriptive data for El Salvador and Nicaragua

		El Salvador				Nicaragua			
		CKD admissions^a^/Inhabitant^b^	EE^c^	Municipalities		CKD deaths/NCD deaths	EE^d^	Municipalities	
				N	%			N	%
Population density quartile	1	3788/618530	ref	65	25%	311/2712	ref	38	25%
	2–3	18,298/2574888	1.2	130	50%	1154/6972	1.4	76	50%
	4	8937/2550695	0.6	67	25%	1964/14607	1.2	39	25%
Crop and cultivation intensity	T_max_								
Banana									
Low	< 30°	13,933/3897281	ref	163	62%	992/14380	ref	93	61%
	> 30°	5515/794353	1.9	47	18%	1819/6055	4.4	30	20%
High	< 30°	1915/297710	1.8	20	8%	140/2791	0.7	27	18%
	> 30°	9778/754769	3.6	32	12%	477/1065	6.5	3	2%
Rice									
Low	< 30°	13,417/3603089	ref	140	53%	843/14044	ref	97	63%
	> 30°	13,778/1333010	2.8	70	27%	2091/5018	6.9	26	17%
High	< 30°	2431/591902	1.1	43	16%	289/3127	1.5	23	15%
	> 30°	1515/216112	1.9	9	3%	205/2102	1.6	7	5%
Sugarcane									
Low	< 30°	11,383/2865554	ref	146	56%	864/14921	ref	104	68%
	> 30°	9245/1263238	1.8	64	24%	602/3648	2.8	19	12%
High	< 30°	4465/1329437	0.8	37	14%	268/2250	2.1	16	10%
	> 30°	6048/285884	5.3	15	6%	1694/3472	8.4	14	9%
Coffee									
Low	< 30°	8834/2261478	ref	137	52%	963/13977	ref	91	59%
	> 30°	15,088/1472670	2.6	73	28%	2294/7079	4.7	32	21%
High	< 30°	7014/1933513	0.9	46	18%	169/3194	0.8	29	19%
	> 30°	205/76452	0.7	6	2%	2/41	0.7	1	1%

^a^Number of cases based on multiplying municipal rates in 2005–2010 [6] with the population in 2007 [25]. ^b^Population in 2007 [25]. ^c^Admissions ratio, ^d^Proportional mortality ratio. *CKD* Chronic Kidney Disease, *NCD* Non-communicable Disease, *EE* effect estimate

**Fig. 1:**
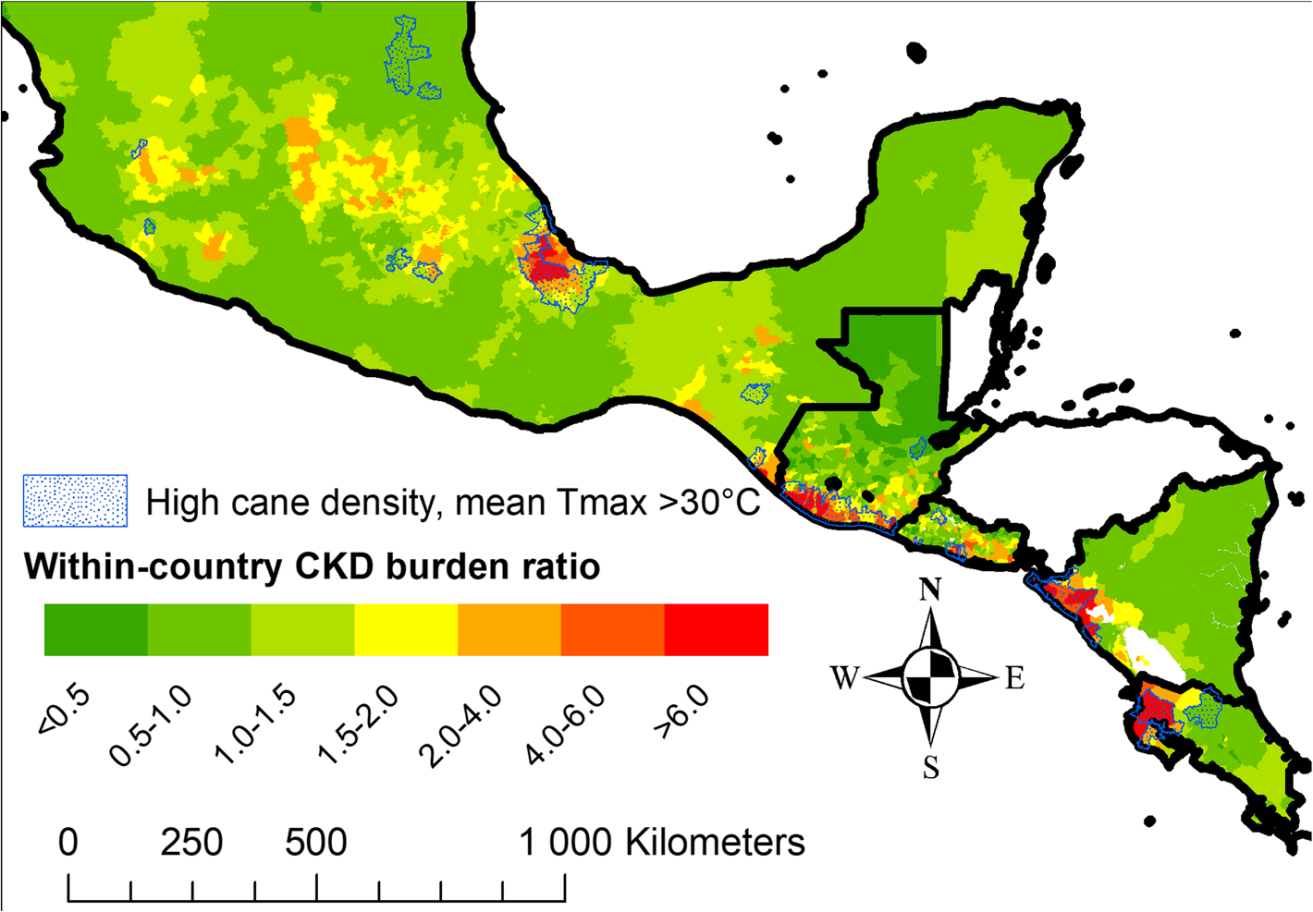
Within-country CKD burden ratio. CKD burden ratio: CKD mortality rate ratio in working age men for Mexico, Guatemala and Costa Rica. Proportion odds ratio for CKD deaths out of total non-communicable disease deaths in all age and sex groups in Nicaragua. Admission rate ratio for unspecified CKD for all age and sex groups in El Salvador. Geographical units are second-level administrative units, subnational borders are not shown. > 30 °C Mean maximum temperature according to Worldclim.org [27]. High cane density is top 20% municipalities in each country except for Mexico, for which it is the top 5%. The map is adapted from the Database of Global Administrative Areas (https://gadm.org/), with permission

The CKD burden, i.e. the proportional mortality odds ratio (Nicaragua) or mortality ratio (Guatemala, Mexico, Costa Rica), or hospital admissions rate ratio (El Salvador), varied markedly within all countries (Fig. 1), with high burden primarily along the Pacific coast, but also in Veracruz on the Mexican Gulf coast.

**Fig. 2:**
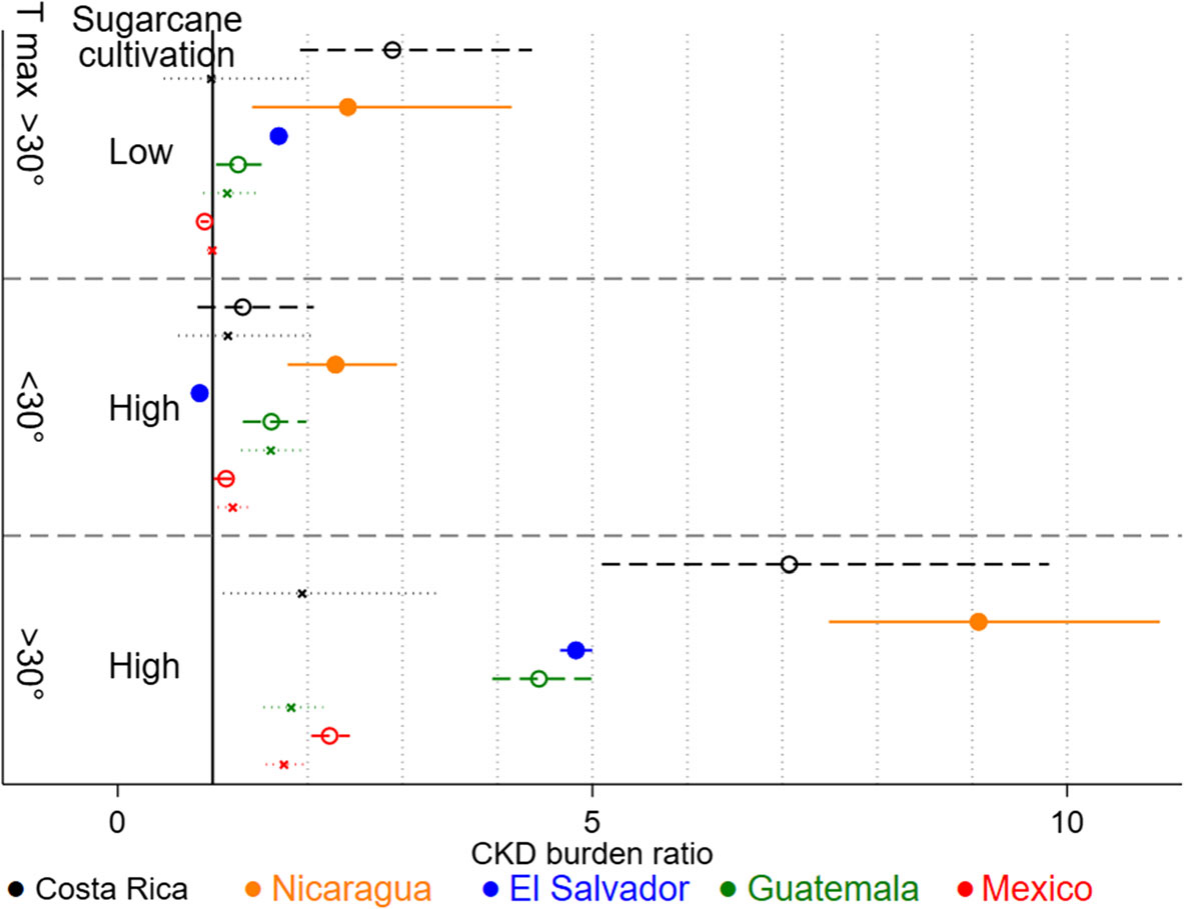
Within-country CKD burden ratio by sugarcane-heat category adjusted for population density. CKD burden ratio: CKD mortality rate ratio in working age men (dashed) and women (dotted) for Mexico, Guatemala and Costa Rica. Proportion odds ratio for CKD deaths out of total non-communicable disease deaths in all age and sex groups in Nicaragua. Admission rate ratio for unspecified CKD for all age and sex groups in El Salvador. T_max_ = Mean maximum temperature according to Worldclim.org [27]

There was a trend towards increasing CKD burden at yearly mean maximum temperatures above rather than below 30 °C. Sugarcane was the only crop which in all countries was associated with a higher CKD burden when intensely rather than sparsely cultivated in such heat (CKD burden ratios at least doubling in all countries, increasing from approximately 1–3 to 2–9 in all countries), but there was no such clear CKD burden increase when sugarcane was intensely cultivated in less hot regions (CKD burden ratios at 1–2 in all countries) (Fig. 2). A similar pattern remained in spatial regression models although the effect estimates were reduced and the credibility intervals obtained through these Bayesian regression models were wider than the confidence intervals obtain from the non-spatial frequentist models (Supplement C; Figure C2). For countries from which sex-stratified analyses were possible (Mexico, Guatemala and Costa Rica), the pattern of association with sugarcane in heat was more pronounced for men than women.

High intensity cultivation of coffee, rice and banana was not consistently associated with increased CKD burden, and there was no evident increase in CKD burden when any of these crops were intensely cultivated above 30 °C (Supplement C; Figure C1 and C3).

## Discussion

By using geostatistical tools to reduce the influence of random fluctuations and unveil the underlying heterogeneous risk surface this study identified areas within each Mesoamerican country with markedly elevated CKD burden in routinely collected data. Geostatistical tools also enabled us to examine associations between outcome and exposure, in this case identifying that sugarcane cultivation in high heat consistently is associated with elevated CKD burden.

The spatial variation in CKD burden throughout Mesoamerica largely follows the expected pattern of higher disease burden along the Pacific coast, as has been reported previously in country-specific studies of Costa Rica [3], El Salvador [6] and Guatemala [4]. This study adds a quantitative analysis of all countries, higher resolution than previously presented, and data from Nicaragua and Mexico. While most hotspots are on the Pacific coast one area is also identified on the Mexican Caribbean coast, a hot sugarcane cultivation area close to a suspected CKDnt hotspot [37]. While we could not access data from Belize, a survey report higher CKD prevalence in the northern part [38], a hot region which is the primary sugarcane cultivation area in that country [39]. Likewise, in Panama high CKD mortality is reported from the hot lowland sugarcane-cultivating Coclé province [40].

The association between CKD burden and intense sugarcane cultivation in heat is expected considering several individual-level studies finding that sugarcane workers who perform strenuous activity in heat have a high risk of kidney injury and CKDnt [19]. The stronger association in men than women suggests an occupational rather than environmental exposure, consistent with men more often performing the physically most strenuous tasks in sugarcane cultivation [41, 42]. However, we cannot rule out biological differences influencing susceptibility to the same exposure. Our findings thus support etiological hypotheses of repeated heat stress as a cause of CKDnt. The lack of consistent associations between cultivation of banana, coffee, or rice, and CKD imply that work practices or agrochemicals used primarily for cultivation of these crops are not key causal factors for CKDnt initiation or progression. Thus, future studies exploring the differences in occupational exposures and living conditions in communities near these crops and sugarcane could point to important causal mechanisms.

Importantly, some areas that are not very hot sugarcane cultivation areas also have an elevated CKDnt burden and vice versa (e.g. Mexico in Fig. 1), and these could be especially interesting to consider for further field studies. CKD does however not equal CKDnt. Subnational variations in traditional risk factors such as diabetes or hypertension, which may be linked to urbanization, is one possibility. However, in a secondary analysis, exclusion of the municipalities in the highest population density category accentuated the gradient between sugarcane and heat and CKD in four of the five countries. Other spatially correlated kidney disease entities may exist in parallel with CKDnt in Mesoamerica. As one example there are reports of albuminuric kidney disease, possibly caused by a toxin, around Lake Chapala in western Mexico [43], and this area is highlighted in our analysis.

This is an ecological study meaning findings do not necessarily hold at an individual level. It may be that it is not the sugarcane workers in hot areas that suffer from CKD, but another group which often happens to reside in the same municipality. Considering this design and use of routine data not collected for research use, this study should be seen as hypothesis generating/prioritizing rather than hypothesis testing.

Administrative problems leading to incorrect data cannot be ruled out. For El Salvador, the use of hospital admissions data means that e.g. socio-economic differences influencing access to healthcare may influence the results. Occupational exposure in distant municipalities is another source of potential source of error and bias, as much agricultural work is conducted by migrant workers, and prevalence surveys should carefully consider migratory work. As an example, since decades ago much sugarcane harvesting in Costa Rica has been done by migrant workers, mostly from Nicaragua (Catharina Wesseling, personal communication). Crops that employ a more significant proportion of the workers in a municipality may have a larger influence on the risk for that municipality than a crop that employs fewer, meaning signals for the latter crop will be weaker. The proportion of the workforce employed for cultivation of each type of crop should ideally have been considered as exposure variable rather than the area or tons harvested, but such information was not available.

Globally smooth random effects structures, such as the one used for spatial regression modelling, have been criticized for attenuating risk estimates for spatially correlated covariates as these correlate with the spatial random effects structure, making it difficult to identify the contribution of each [35, 36]. Spatial regression models with localized conditional autoregressive priors have been developed to allow for step changes in the risk surface [35], but these methods are more complex and not within the scope of this study. Higher estimates when not including spatially correlated random effects suggest the spatially adjusted risk estimate for the sugarcane-heat covariate may be attenuated in the spatial model. Hot cane-cultivating municipalities are often confined to one or a few single continuous areas per country and thus strongly spatially correlated, making it difficult to differentiate an association with sugarcane cultivation in heat from an unmeasured, spatially correlated risk factor confined to that area using spatial regression models.

Thus, an interpretation of the estimates of the non-spatial model should consider that it is possible that effects may be overestimated and confidence intervals too narrow due to spatial autocorrelation, while an interpretation of the spatial model estimates should consider that effects could possibly be underestimated due to attributing too much importance to unmeasured spatially correlated factors. The “true” effect estimate is most likely to be somewhere between the estimates of the spatial and non-spatial models. A literal interpretation of the model output is not especially meaningful, both considering this analytical aspect, but arguably also considering the study design. Rather, we argue that the relative strength of the estimate for different crop-heat combinations, and the consistency of these patterns between countries is of interest for approaching a discussion on causality. Consistent patterns in all five countries make a causal relationship between sugarcane cultivation in heat and CKDnt more likely, especially considering several individual-level studies documenting a high risk of acute kidney injury and chronic kidney disease among sugarcane workers [19, 41, 42].

Maximum ambient temperature (T_max_) does not account for humidity, solar radiation or wind speed, factors which are additional important determinants of heat stress, especially among outdoor workers. Use of the wet-bulb globe temperature (WBGT) [44] would have been superior, but such measurements are not available and as far as we are aware, WBGT estimates based on available climate data has only been elaborated for indoor conditions [45]. Estimation of outdoor WBGT is needed to better understand the impact of heat on agricultural workers, especially considering rising global temperatures. Seasonal variation in heat and agricultural activity (e.g. sugarcane harvest season) is not accounted for in the present analysis. More knowledge about seasonally correlated activities performed for each crop, and how relatively physically intense these are, in each country, and ideally also within countries, would have been needed to account for this, but this was considered outside of the scope of this study.

Information on area-level agrochemical use, which includes information on the specific substances used, does to our knowledge not exist in Mesoamerica. California has detailed records on pesticide use [46] and CKD [47] and connecting these data sources could be valuable for detecting potentially nephrotoxic pesticides.

Death from CKD likely occur several years after exposure to the agent leading to kidney damage, why it would have been most relevant to use historic data on crop cultivation. We accessed mostly crop cultivation data concurrent with outcome data, which is a limitation of this study. Apart from the transition from cotton to sugarcane in some areas in the last decades of the 20th century [33, 34], it is unlikely that there have been major shifts in crop localizations; thus we can reasonably assume that current crop distribution maps relatively well reflect historic exposure over a few decades. Testing this assumption would, apart from reliable data on disease progression rate, need intense efforts to locate historic data, which are unlikely to be easily available in digitized format at sufficient quality. We do not include non-agricultural sources of occupational heat exposure such as brickmaking, which has been found to be associated with high CKDnt prevalence [48], and spatial clustering of such activities may influence the results provided sufficient proportions of the population are exposed.

The high awareness of the CKDnt epidemic in affected parts of the Mesoamerican countries means clinicians may be more likely to attribute deaths to CKD in these parts, especially as CKD may be a contributing cause of deaths due to other more direct causes, such as cardiovascular. This may bias towards increased spatial clustering and possibly increased association with explanatory factors.

While a statistical approach with a combined analysis of all countries could have given more condensed estimates of the risk associated with each crop at specific temperatures, there are also inherent difficulties in such analysis. How to appropriately weigh the risk factor estimate from each country is not straightforward, and a combined estimate would be largely uninterpretable, especially considering the different types of data with different quality from each country. We recognize that our graphical approach provides some room for subjective assessment on the association between heat, crops and CKD, but by considering the strength (magnitude of risk elevation) and consistency (between countries) we consider that the association is described in a more transparent way than possible through further advanced statistical modelling, which would incur a false sense of objectivity and precision.

## Conclusion

There are large differences in reported CKD burden within Mesoamerican countries, a variation which at least partially is explained by differences in heat and crop cultivation. The burden is generally highest in hot parts of the countries intensely cultivating sugarcane, in agreement with a large body of literature to date. In contrast, no consistent associations were found for other major agroindustrial crops like coffee, rice and banana. Thus, we found no support for agricultural pesticide use in general as a risk factor. In summary, heat stress in industrial agriculture dependent on strenuous manual labour should remain a priority for further studies on how to stop the CKDnt epidemic.

## Supplementary Information

**Additional file 1: Supplement A.** Handling exposure variables in ArcGIS.

**Additional file 2: Supplement B.** Spatial regression modelling.

**Additional file 3: Supplement C.** Additional results.

## Data Availability

Datasets generated and/or analyzed during the current study are available from the corresponding author on reasonable request and if the consent of the original data holder can be obtained. Individual level data (completely anonymized) from Mexico was downloaded from a governmental website [21]. Individual level data (completely anonymized) from Guatemala was downloaded from a governmental website [22] Area level data from Nicaragua was collected from a governmental website [23]. Area level data from El Salvador [6] was obtained from the corresponding author since the published link to data was broken. Area level data from Costa Rica [3] was obtained via the corresponding author. Please see Table 2 for references to datasets from which the explanatory variables were obtained.

## Abbreviations

CKD: Chronic Kidney Disease
CKDnt: Chronic kidney disease of non-traditional origin
MINSA: Ministerio de Salud de Nicaragua
INEC: Instituto Nacional de Estadistica y Censo Costa Rica
WBGT: Wet-bulb globe temperature
T_max_: Monthly maximum ambient temperature
