# Supplementary material for: An ecological study of chronic kidney disease in five Mesoamerican countries: associations with crop and heat
Source: BMC Public Health. 2021 May 1;21:840. doi: 10.1186/s12889-021-10822-9 (PMC8088703; doi:10.1186/s12889-021-10822-9)
Supplement: Supplementary file 1 — Additional file 1: Supplement A. Handling exposure variables in ArcGIS. [file 12889_2021_10822_MOESM1_ESM.docx]

# Supplement A: Handling exposure variables in ArcGIS

ArcGIS was used for linking spatially defined exposure variables to municipalities by extracting T_max_, population density and crop intensity (below) raster layers to the municipality centroid.

#### Development of crop cultivation intensity raster layers

Neither occupational nor environmental exposure to a certain crop cultivation must be strictly limited to municipality borders. Workers may work in cultivation of a crop in a neighboring municipality although there is no cultivation of the crop in the municipality in which they reside, and agrochemicals may drift from one municipality to another one nearby. We sought to make a more realistic exposure assessment by allowing municipalities near intense cultivation of a specific crop also to be considered exposed to this.

This was achieved by randomly allocating points within each municipality (or sub-municipal area used for cultivation of this crop when available (Nicaragua and Costa Rica for sugarcane)). The number of points placed in each municipality/area was proportional to the tons or acres harvested of each specific crop per municipality as reported by national statistics or agricultural ministries. The municipality in each country with the most production or acreage of a certain crop (e.g. coffee) was assigned 200 points, a hypothetical municipality with exactly half production/acreage of the municipality with most production/acreage was assigned 100 points, and so forth until municipalities with less than 1/400^th^ of that production/acreage were assigned 0 points, for that specific crop. For Guatemala, where information on production or acreage for each crop was unavailable, a detailed shape format land use map provided high-resolution information on where and how large cultivations there were of sugarcane, coffee, and banana and points were allocated within these polygons, with a number proportional to their size. Rice was only available at department level for Guatemala, hence this procedure was done at the department level for rice in Guatemala.

Once these crop points had been allocated, the Point Density tool was used to create a raster of how many points there were within 0.18 decimal degrees (approximately 20 km). This distance was chosen as unpublished data from a sugarcane worker cohort at a Nicaraguan mill (1) indicate that most workers live within this distance from the mill, indicating that this is a common commuting distance. The value of this raster was extracted to the municipality centroid points.


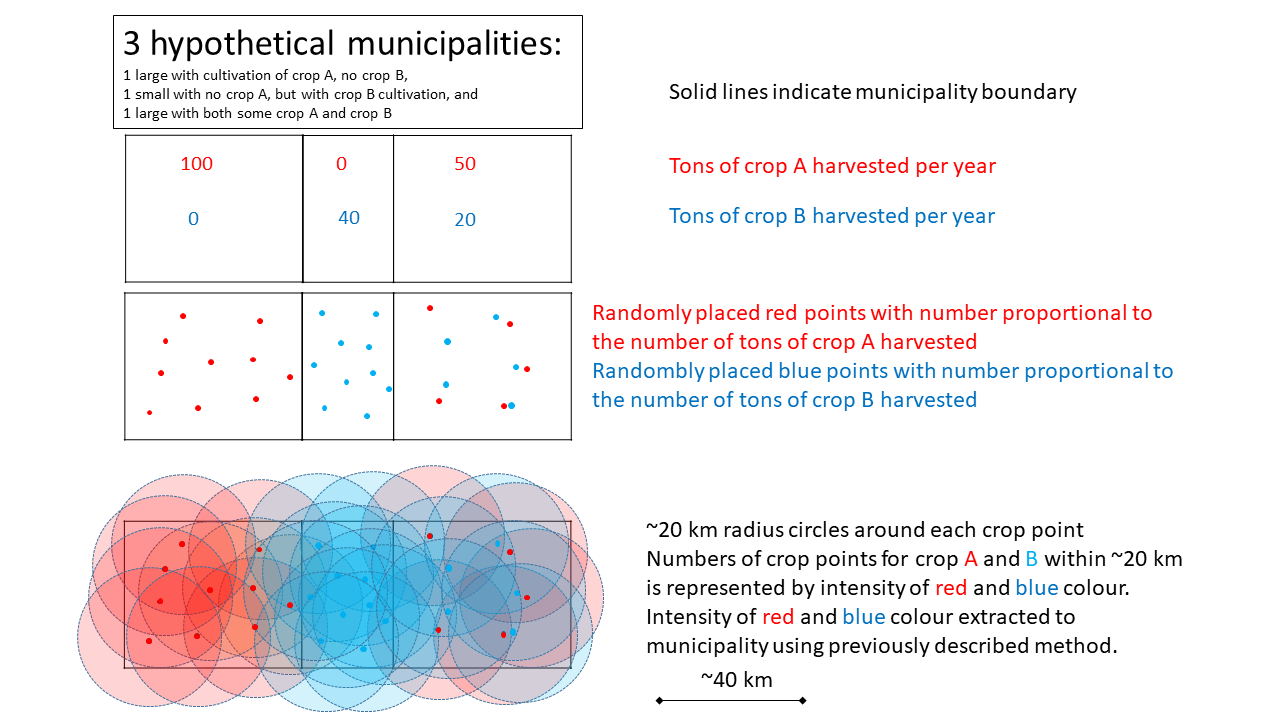


Figure 1. Description of the method used to assess crop cultivation intensity

## References

1. Hansson E, Glaser J, Weiss I, Ekström U, Apelqvist J, Hogstedt C, et al. Workload and cross-harvest kidney injury in a Nicaraguan sugarcane worker cohort. Occupational and environmental medicine. 2019;76(11):818-26.
