# Supplementary material for: An ecological study of chronic kidney disease in five Mesoamerican countries: associations with crop and heat
Source: BMC Public Health. 2021 May 1;21:840. doi: 10.1186/s12889-021-10822-9 (PMC8088703; doi:10.1186/s12889-021-10822-9)
Supplement: Supplementary file 2 — Additional file 2: Supplement B. Spatial regression modelling. [file 12889_2021_10822_MOESM2_ESM.docx]

# Supplement B: Spatial regression modelling

## Model specification

For all models, we modelled the aggregated number of observed outcome events in the time period for which there was data. The expected number of outcome events was based on the population size for the year which was most central in the period for which outcome data was available. If for example outcome data was available for 2004-2012 and population data was available for 2005 and 2010, then 2010 population data was used.

### Regression model for mapping

The following models were used to smooth mortality rate estimates to obtain CKD burden estimates for mapping (Figure 1).

#### Guatemala, Mexico and Costa Rica

${Observed CKD deaths}_{i} \sim Poisson\left( \mu_{i} \right)$ Equation 1 A

$\mu_{i}=\theta_{i}\times Expected{CKD deaths}_{i}$ Equation 1 B

$\log\left( \theta_{i} \right)=\propto+ \omega_{i}+\vartheta_{i}+$ Equation 1 C

${CKD burden ratio}_{i}=exp\left( \omega_{i}+\vartheta_{i} \right)$ Equation 1 D

#### El Salvador

${Observed unspecified CKD admissions}_{i} \sim Poisson\left( \mu_{i} \right)$ Equation 2 A

$\mu_{i}=\theta_{i}\times Expected{unspecified CKD admissions}_{i}$ Equation 2 B

$\log\left( \theta_{i} \right)=\propto+ \omega_{i}+\vartheta_{i}$ Equation 2 C

${CKD burden ratio}_{i}=exp\left( \omega_{i}+\vartheta_{i} \right)$ Equation 2 D

#### Nicaragua

${Observed CKD deaths}_{i} \sim Binomial\left( {Observed NCD deaths}_{i},p_{i} \right)$ Equation 3 A

$\mathrm{logit} \left( p_{i} \right)=\propto+ \omega_{i}+\vartheta_{i}$ Equation 3 B

${CKD burden ratio}_{i}=exp\left( \omega_{i}+\vartheta_{i} \right)$ Equation 3 C

Expected number of CKD deaths was calculated as the age-standardized (5-year age bands). For El Salvador only the population size was used to calculate estimated number of CKD admissions due to no information on the age of CKD patients.

CKD, Chronic Kidney Disease. NCD, Non-communicable disease (includes CKD).

*i* denotes municipality*.*

$\propto$ is the intercept, specified using WinBUGS *dflat()* uniform distribution, i.e. with all values equally likely.

$\omega_{i}$ is a spatially uncorrelated random effect modelled using an unstructured normally distributed prior with mean 0 and a precision (i.e. 1/variance) specified using a gamma distribution with shape and rate parameter set to 0.001.

$v_{i}$ is a spatially correlated random effect modelled using a Gaussian conditional autoregressive (CAR) prior structure (1), using an adjacency matrix with adjacent municipalities given a weight of one and non-adjacent given a weight of zero. Again, the prior for the precision of the Gaussian CAR random effect was specified by a gamma distribution with shape and rate parameter of 0.001.

Including both a spatially correlated and uncorrelated random effect is a common choice in spatial epidemiology (2), and it allows accounting for variation both explained by the unmeasured risk factors in the area and surrounding areas, and unmeasured factors that may be unique to the one single area, i.e. on a sub-area scale.

For Nicaragua, only the top 15 mortality causes per year was available for each municipality and there was no age and sex information. Therefore, in Nicaragua we assumed the CKD mortality rate in municipalities reporting no CKD deaths and more than 15 different causes of death was the same as in the largest municipality. For Nicaragua we modelled the proportion CKD deaths out of other reported non-communicable disease (NCD) deaths using logistic regression to account for differences in age structure.

### Spatial regression modelling for crop-heat association analysis

The following models were used to assess the association between the interaction of crop cultivation intensity, heat and CKD burden, adjusting for population density, while accounting for the spatial structure of the data.

#### Guatemala, Mexico and Costa Rica

${Observed CKD deaths}_{i} \sim Poisson\left( \mu_{i} \right)$ Equation 7 A

$\mu_{i}=\theta_{i}\times Expected{CKD deaths}_{i}$ Equation 7 B

$$\log\left( \theta_{i} \right)=\propto+ \omega_{i}+\vartheta_{i}+ \beta_{1}*{low population density}_{i}+\beta_{2}*{high population density}_{i}+\beta_{3}*{low crop density>30^{\circ}}_{i}+\beta_{4}*{high crop density<30^{\circ}C}_{i}+ \beta_{5}*{high crop density>30^{\circ}}_{i}$$

Equation 7 C

${CKD burden ratio}_{i}=exp\left( \omega_{i}+\vartheta_{i} \right)$ Equation 7 D

#### El Salvador

${Observed unspecified CKD admissions}_{i} \sim Poisson\left( \mu_{i} \right)$ Equation 8 A

$\mu_{i}=\theta_{i}\times Expected{unspecified CKD admissions}_{i}$ Equation 8 B

$\log\left( \theta_{i} \right)=\propto+ \omega_{i}+\vartheta_{i}+ \beta_{1}*{low population density}_{i}+ \beta_{2}*{high population density}_{i}+\beta_{2}*{low crop density>30^{\circ}}_{i}+\beta_{3}*{high crop density<30^{\circ}C}_{i}+ \beta_{4}*{high crop density>30^{\circ}}_{i}$ Equation 8 C

${CKD burden ratio}_{i}=exp\left( \omega_{i}+\vartheta_{i} \right)$ Equation 8 D

#### Nicaragua

${Observed CKD deaths}_{i} \sim Binomial\left( {Observed NCD deaths}_{i},p_{i} \right)$ Equation 9 A

$\mathrm{logit} \left( p_{i} \right)=\propto+\omega_{i}+\vartheta_{i}+\beta_{1}*{low population density}_{i}++\beta_{2}*{high population density}_{i}+\beta_{3}*{low crop density>30^{\circ}}_{i}+\beta_{4}*{high crop density<30^{\circ}C}_{i}+ \beta_{5}*{high crop density>30^{\circ}}_{i}$ Equation 9 B

${CKD burden ratio}_{i}=exp\left( \omega_{i}+\vartheta_{i} \right)$ Equation 9 C

$\beta_{1-5}$ have normal prior distributions with mean 0 and precision 0.01. $\beta_{1-5}$ were translated to the odds or rate ratio scale by exponentiation.

## Model estimation and convergence assessment

Models were estimated by Markov chain Monte Carlo (MCMC) simulations using WinBUGS.

Two Monte Carlo chains with different initial values were run 100000 times and convergence was then assessed. The convergence of the parameters was assessed by inspecting that the chains mixed adequately, indicating that they sample from a stable posterior distribution. Interpretation was aided by calculating the Gelman-Rubin convergence statistic as modified by Brooks and Gelman (3) and displaying these statistics graphically using this function in WinBUGS.

Parameters of interest (municipality-level CKD burden ratio and risk factor coefficient estimates) quickly reached convergence, while variance parameters were slower. Identification of the spatially correlated versus uncorrelated component of the random effect structure is often difficult (2). This seems to be the case also in our data as these different components of the random effects are negatively correlated in the MCMC chains, and can explain slow convergence for these parameters.

The primary interest of this study is to describe where high-risk areas are and risk factors associated with these. It is not to determine whether the spatial variation is on the individual municipality level or if there is a spatial dependence between nearby municipalities. Thus, we consider it of less importance that we cannot separate spatially structured and unstructured variation. The purpose is that spatial dependence due to unmeasured risk factors is accounted for in the model, and allowing for both correlated and uncorrelated random effects should do so (2).

## References

1. Besag J, York J, Mollié A. Bayesian image restoration, with two applications in spatial statistics. Annals of the Institute of Statistical Mathematics. 1991;43(1):1-20.

2. Lawson AB. Bayesian Disease Mapping. 3 ed: Chapman & Hall/CRC; 2018.

3. Brooks S, Gelman A. General Methods for Monitoring Convergence of Iterative Simulations1998. 434-55 p.
