# Supplementary material for: An ecological study of chronic kidney disease in five Mesoamerican countries: associations with crop and heat
Source: BMC Public Health. 2021 May 1;21:840. doi: 10.1186/s12889-021-10822-9 (PMC8088703; doi:10.1186/s12889-021-10822-9)
Supplement: Supplementary file 3 — Additional file 3: Supplement C. Additional results. [file 12889_2021_10822_MOESM3_ESM.docx]

# Supplement C: Additional results


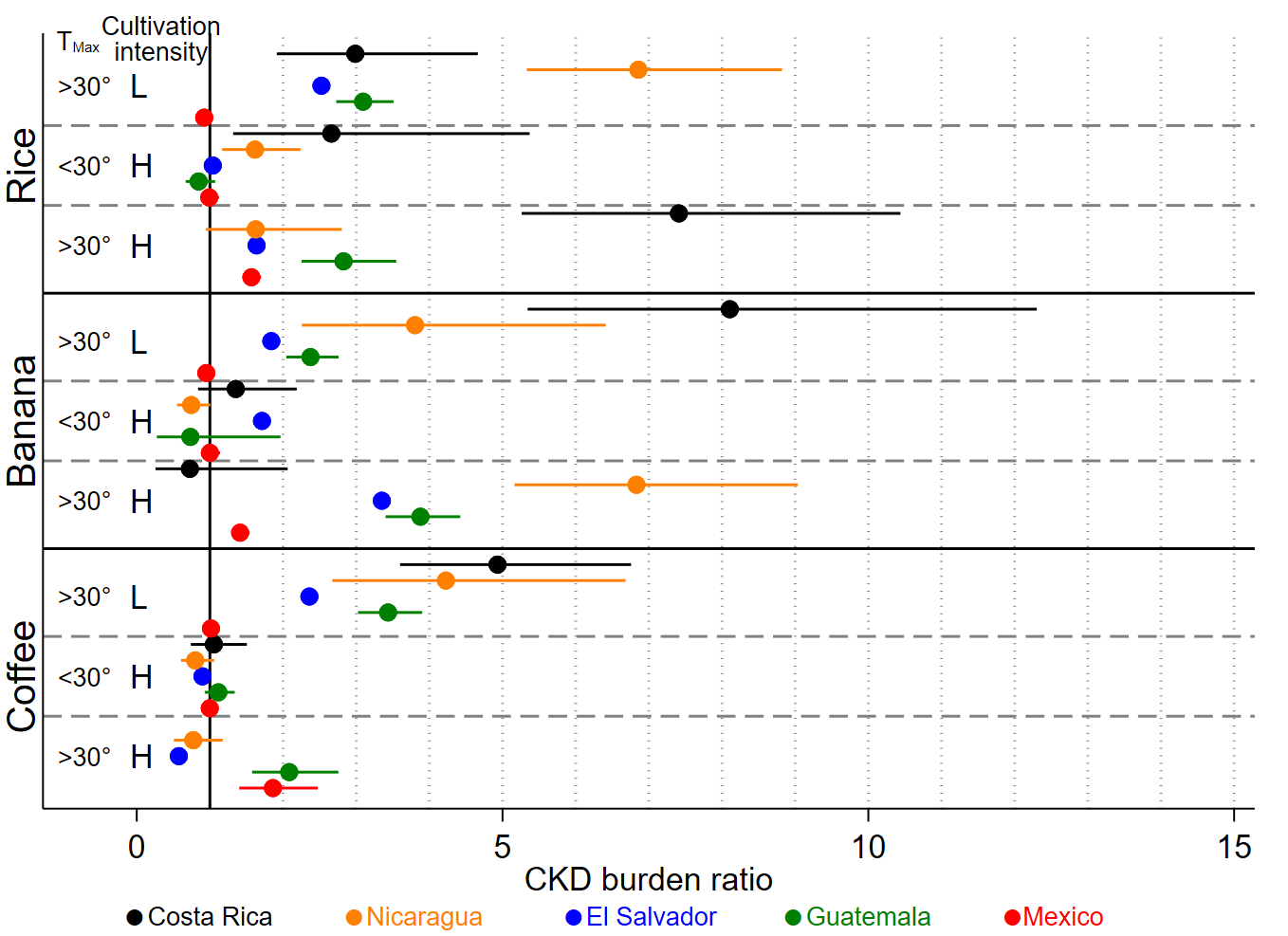


Figure C1 Association between CKD, crop (coffee, rice and banana) and heat by country in non-spatial regression analysis adjusted for population density.

CKD burden ratio= CKD mortality rate ratio in working age men for Mexico, Guatemala and Costa Rica. Proportion odds ratio for CKD deaths out of total non-communicable disease deaths in all age and sex groups in Nicaragua. Admission rate ratio for unspecified CKD in all age and sex groups in El Salvador. CKD=Chronic Kidney Disease. T_max_=Mean maximum temperature according to Worldclim.org (1,2). L=Low cultivation intensity. H=High cultivation intensity


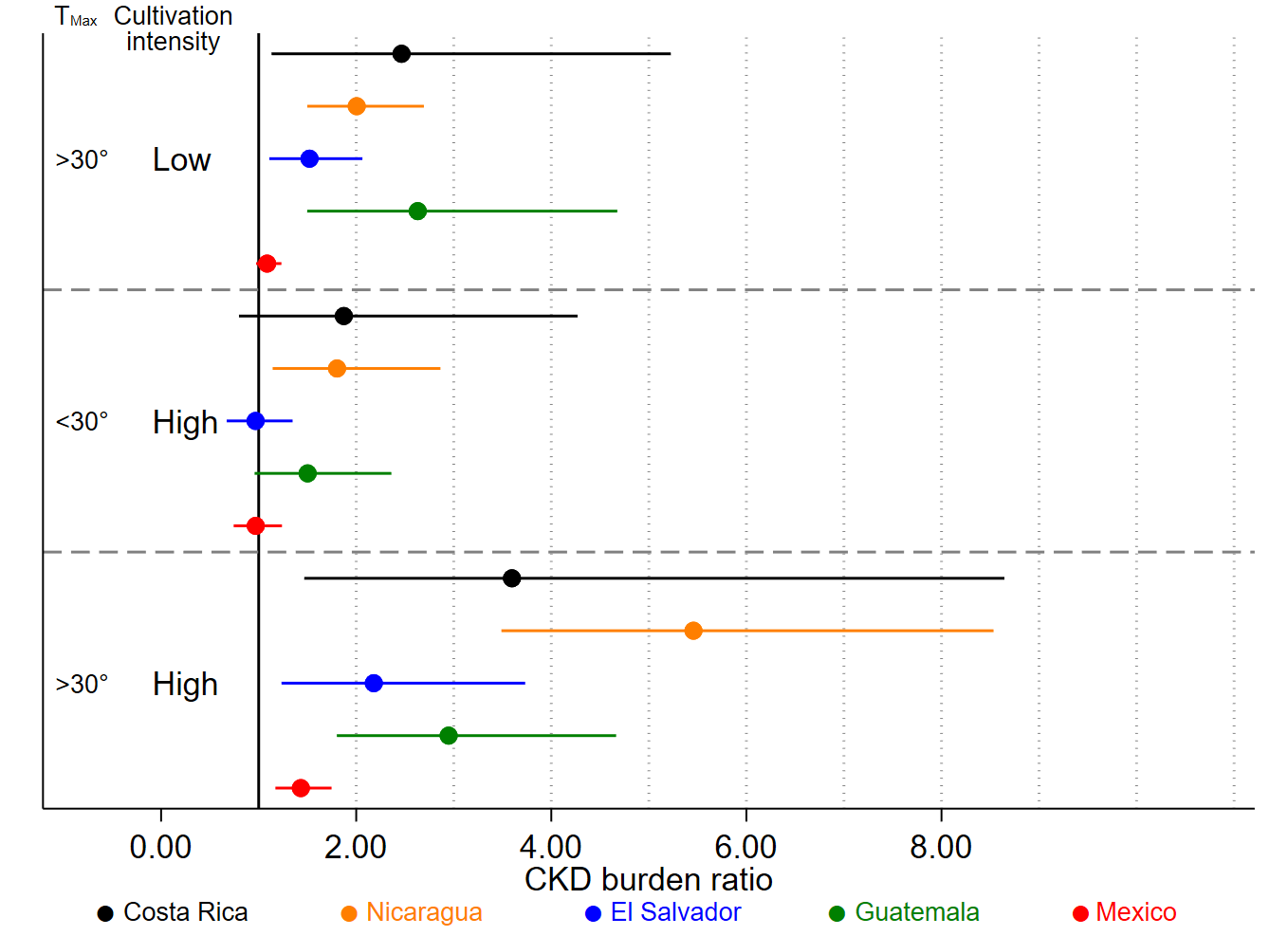


Figure C2 Association between CKD, sugarcane cultivation and heat by country in spatial regression analysis adjusted for population density.

CKD burden ratio= CKD mortality rate ratio in working age men for Mexico, Guatemala and Costa Rica. Proportion odds ratio for CKD deaths out of total non-communicable disease deaths in all age and sex groups in Nicaragua. Admission rate ratio for unspecified CKD in all age and sex groups in El Salvador. CKD=Chronic Kidney Disease. T_max_=Mean maximum temperature according to Worldclim.org (1,2).


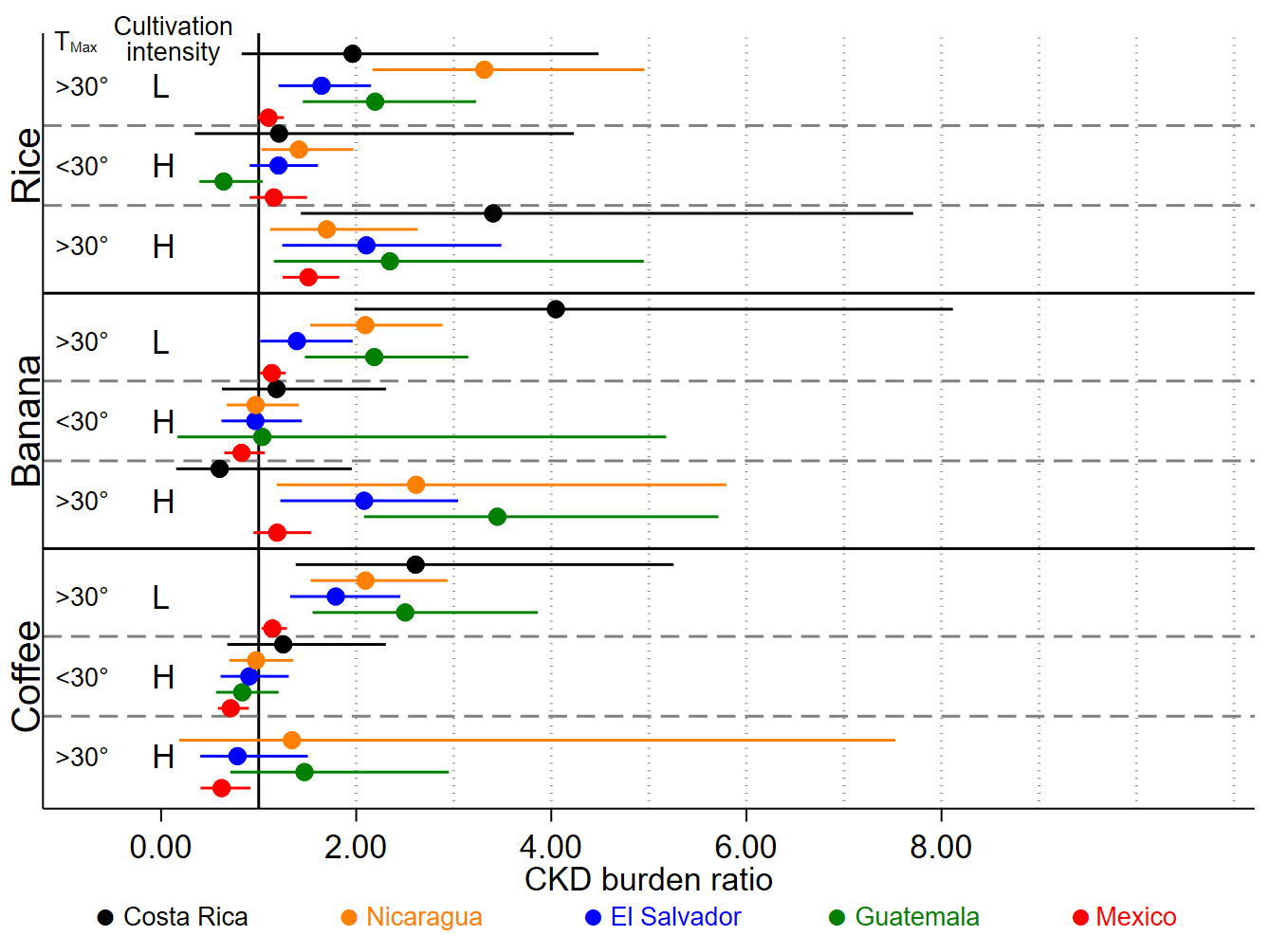


Figure C3 Association between CKD, crop and heat by country in spatial regression analysis adjusted for population density.

CKD burden ratio= CKD mortality rate ratio in working age men for Mexico, Guatemala and Costa Rica. Proportion odds ratio for CKD deaths out of total non-communicable disease deaths in all age and sex groups in Nicaragua. Admission rate ratio for unspecified CKD in all age and sex groups in El Salvador. CKD=Chronic Kidney Disease. T_max_=Mean maximum temperature according to Worldclim.org (1,2). L=Low cultivation intensity. H=High cultivation intensity

## References

1. Fick SE, Hijmans RJ. WorldClim 2: new 1-km spatial resolution climate surfaces for global land areas. International Journal of Climatology. 2017;37(12):4302-15.

2. WorldClim - Global Climate Data, <http://worldclim.org/version2>. Accessed 2018-04-06
